# Protective Effect of SeMet on Liver Injury Induced by Ochratoxin A in Rabbits

**DOI:** 10.3390/toxins14090628

**Published:** 2022-09-08

**Authors:** Ziqiang Zhang, Jingyi Xu, Xin Zhang, Jiajia Wang, Hui Xie, Yingying Sun, Qianwen Zhang, Zhaoyang Chang, Yumei Liu

**Affiliations:** College of Animal Science and Technology, Henan University of Science and Technology, Luoyang 471000, China

**Keywords:** Ochratoxin A (OTA), Nrf2, oxidative stress, inflammation, selenomethionine (SeMet)

## Abstract

Ochratoxin A (OTA) is second only to aflatoxin in toxicity among mycotoxins. Recent studies have shown that selenomethionine (SeMet) has a protective effect on mycotoxin-induced toxicity. The purpose of this study was to investigate the protective effect and mechanism of SeMet on OTA-induced liver injury in rabbits. Sixty 35-day-old rabbits with similar body weight were randomly divided into five groups: control group, OTA group (0.2 mg/kg OTA), OTA + 0.2 mg/kg SeMet group, OTA + 0.4 mg/kg SeMet group and OTA + 0.6 mg/kg SeMet group. Rabbits were fed different doses of the SeMet diet for 21 d, and OTA was administered for one week from day 15 (the control group was provided the same dose of NaHCO_3_ solution). The results showed that 0.4 mg/kg SeMet could significantly improve the liver injury induced by OTA poisoning. SeMet supplementation can improve the changes in physiological blood indexes caused by OTA poisoning in rabbits and alleviate pathological damage to the rabbit liver. SeMet also increased the activities of SOD, GSH-Px and T-AOC and significantly decreased the contents of ROS, MDA, IL-1β, IL-6 and TNF-α, effectively alleviating the oxidative stress and inflammatory response caused by OTA poisoning. In addition, OTA poisoning inhibits Nrf2 and HO-1 levels, ultimately leading to peroxide reaction, while SeMet activates the Nrf2 signaling pathway and enhances the expression of the HO-1 downstream Nrf2 gene. These results suggest that Se protects the liver from OTA-induced hepatotoxicity by regulating Nrf2/HO-1 expression.

## 1. Introduction

Mycotoxins are secondary metabolites produced by fungi at appropriate temperatures and humidity. According to the Ministry of Food and Agriculture, up to 25 percent of the world’s food is contaminated with mycotoxins, causing the loss of approximately 1 billion tons of food each year [1]. Mycotoxins enter animals and humans through contaminated crops, causing serious health effects [2]. Ochratoxin A (OTA) is the most common and contaminated mycotoxin, second only to aflatoxin in toxicity [3]. Due to the stable chemical properties of OTA, it can withstand the physical and chemical conditions of modern food processing without degradation [4], and the decomposition and elimination rate of OTA is very slow. It is also difficult to be excreted with metabolism, thus accumulating in animal tissues and organs and endangering health [5,6]. Therefore, OTA will not cause acute diseases in terms of clinical manifestations. Instead, chronic poisoning will be caused as animals and humans consume a small amount of food contaminated by OTA for a long time, showing hepatotoxicity, genotoxicity, immunosuppression, teratogenicity, carcinogenicity and so on [7]. The liver, as the primary metabolic and detoxification organ, is one of the target organs of OTA toxicity. Studies have shown that even low levels of OTA exposure in animals can lead to pathological and functional changes in the liver [8]. The induction of oxidative stress is the main mechanism of OTA toxicity. Oxidative stress is characterized by a reduction in total antioxidant capacity (T-AOC) and the activity of antioxidant enzymes, including catalase (CAT), glutathione peroxidase (GSH-px) and superoxide dismutase (SOD) [9]. Nrf2 gene expression is closely related to cell detoxification, cell protection and antioxidant activity [10]. Studies have shown that OTA is an inhibitor of Nrf2, which inhibits the activation and transcription of Nrf2, leading to the production of ROS, resulting in lipid peroxidation and protein and DNA damage [11].

Antioxidants are reported to protect cells from OTA-induced cytotoxicity and genotoxicity [12,13]. Selenium (Se) has been proved to be an essential trace element for life activities and maintaining health, with functions of promoting immunity, anti-oxidation and anti-cancer [14]. Studies found that Se is the main structural component of many enzymes, such as glutathione peroxide (GSH PX), selenoprotein P (SeLP), thioredoxin reductase (TrxRs) and so on, which play an important role in antioxidant activity [15]. It was found that Se can reduce the levels of lipid peroxidation and oxidative stress in the brain of experimental rats by regulating the activity of GSH PX [16]. Ghorbel et al. found that by supplementing Se, the body could restore antioxidant status, ameliorate lung tissue damage and reduce oxidative stress levels [17]. Se has been paid more and more attention due to its excellent antioxidant ability in organisms. It has now been added to animal feed to boost immunity and prevent many diseases. Se plays the role of feed additives, and its existing forms are mainly divided into organic selenium and inorganic selenium. Compared with inorganic selenium, organic selenium represented by selenomethionine (SeMet) is less toxic, safer, more bioavailable, more effective in biological functions and gradually replaces inorganic selenium. Studies have shown that Se can reduce the toxicity of mycotoxins. The expression of antioxidant proteins such as superoxide dismutase (SOD), catalase (CAT) and g-glutamyl can be indirectly induced by Se, resulting in the protection of organ tissues from oxidative damage and enhancing immune function [18]. In addition, by regulating oxidative stress and p38 phosphorylation, Se can attenuate OTA-induced cytotoxicity and apoptosis [19]. It also protects DNA from oxidative damage by scavenging ROS [20]. Therefore, SeMet can improve the immune function, production performance and reproductive capacity of livestock and it has a good prospect in animal production.

Studies have shown that rabbits have a very sensitive reaction to mycotoxin exposure [21]. To date, no effect of SeMet on OTA-induced liver injury in rabbits has been reported. In this study, the toxicity model of OTA rabbits was established to explore the effects of different doses of SeMet on OTA-induced liver injury and explore its specific protective mechanism.

## 2. Results

### 2.1. Plasma Biochemical Analysis

**Figure 1:**
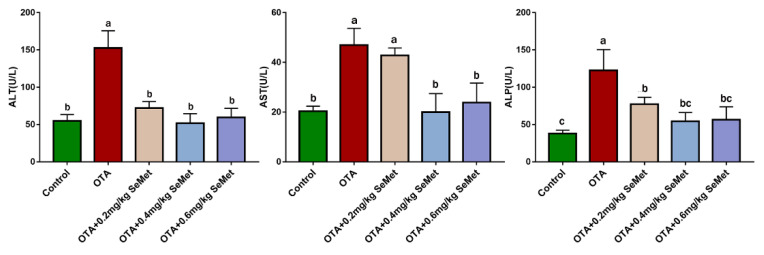
Effects of SeMet at different doses on AST, ALP and ALT levels in serum of rabbits after OTA treatment (Mean ± SD, *n* = 6). ^a–c^ Means column carrying different superscript letters and indicates significant differences (*p <* 0.05).

In order to explore the changes in rabbit liver function in each group, we measured the activities of AST, ALP and ALT in rabbit plasma, which are the key indexes to reflect the changes in liver function. After 7 d of OTA administration, we obtained the results shown in Figure 1. Compared with the control group, the activities of serum ALT, AST and ALP in the OTA group were significantly increased by 1.78, 1.3 and 2.24 times (*p* < 0.05), indicating severe impairment of liver function. Compared with the OTA group, the activities of AST, ALT and ALP in serum were decreased to different degrees after SeMet pretreatment. Among them, the 0.4 mg/kg SeMet + OTA group had the most significant effect, and the activities of alt, AST and ALP were reduced by 66.11%, 57.31% and 55.68%, respectively (*p* < 0.05).

### 2.2. SeMet Improves OTA-Induced Liver Pathological Changes

**Figure 2:**
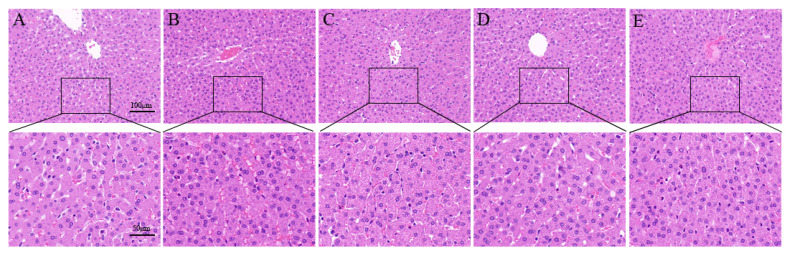
Effects of SeMet on OTA-induced liver morphological changes in rabbits (Mean ± SD, *n* = 6). (**A**) The control group. (**B**) The OTA group. (**C**) The OTA + 0.2 mg/kg Se group. (**D**) The OTA + 0.4 mg/kg Se group. (**E**) The OTA + 0.6 mg/kg Se group. The magnification of the first row of pictures is 200×, and the scale is 100 μm. The second row is a partial magnification of the first row of pictures with a magnification of 400× and a scale of 50 μm.

**Figure 3:**
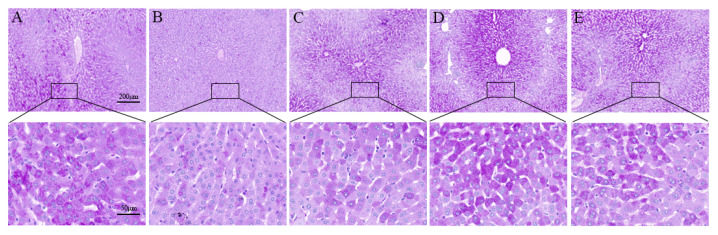
Effect of SeMet on changes of hepatic glycogen content in rabbits induced by OTA (Mean ± SD, *n* = 6). (**A**) The control group. (**B**) The OTA group. (**C**) The OTA + 0.2 mg/kg Se group. (**D**) The OTA + 0.4 mg/kg Se group. (**E**) The OTA + 0.6 mg/kg Se group. The magnification of the first row of pictures is 100×, and the scale is 200 μm. The second row is a partial magnification of the first row of pictures with a magnification of 400× and a scale of 50 μm.

**Figure 4:**
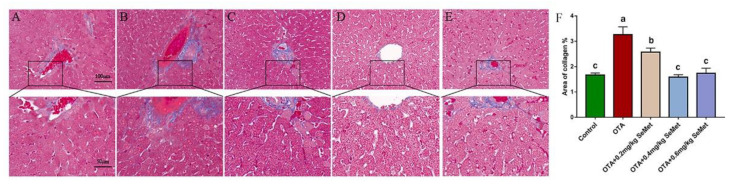
Effect of SeMet on hepatic fibrosis induced by OTA in rabbits (Mean ± SD, *n* = 6). (**A**) The control group. (**B**) The OTA group. (**C**) The OTA + 0.2 mg/kg Se group. (**D**) The OTA + 0.4 mg/kg Se group. (**E**) The OTA + 0.6 mg/kg Se group. (**F**) The statistical results of collagen volume fraction were calculated by ImageJ software. ^a–c^ Means column carrying different superscript letters and indicates significant differences (*p* < 0.05). The magnification of the first row of pictures is 200×, and the scale is 100 μm. The second row is a partial magnification of the first row of pictures with a magnification of 400× and a scale of 50 μm.

Histopathological analysis was performed using H&E, PAS and MASSON staining of the liver sections. In the liver of the control rabbits, liver cells and hepatic sinusoids were radically arranged around the central vein with complete structure, large and round nuclei (Figure 2A), a large amount of liver glycogen (Figure 3A) and the degree of hepatic fibrosis was mild (Figure 4A). Compared with the control group, the OTA-treated group showed unclear contour and disordered arrangement of liver cells, severe vacuolation of cytoplasm, a large number of red blood cells in the hepatic sinus space (Figure 2B), significant reduction of glycogen (Figure 3B) and significantly increased degree of liver fibrosis (Figure 4B). After SeMet pretreatment, pathological changes were improved to varying degrees. In the 0.4 mg/kg SeMet + OTA group, the cell structure of liver tissue was intact, cytoplasmic vacuoles decreased, the number of red blood cells decreased (Figure 2D), hepatic glycogen synthesis increased significantly (Figure 3D) and the degree of hepatic fibrosis decreased significantly (Figure 4D).

### 2.3. SeMet Suppresses OTA-Induced Liver Oxidative Stress

**Figure 5:**
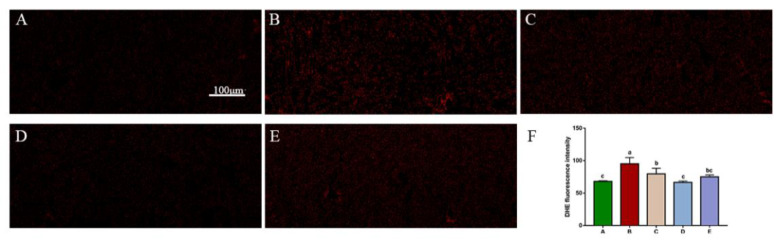
ROS expression in liver. The intensity of the red fluorescence reflects the level of ROS (Mean ± SD, *n* = 6). (**A**) The control group. (**B**) The OTA group. (**C**) The OTA + 0.2 mg/kg Se group. (**D**) The OTA + 0.4 mg/kg Se group. (**E**) The OTA + 0.6 mg/kg Se group. (**F**) Quantitative analysis of fluorescence intensity of DHE staining using ImageJ software. ^a–c^ Means column carrying different superscript letters and indicates significant differences (*p <* 0.05). Magnification is 200×, and scale is 100 μm.

**Figure 6:**
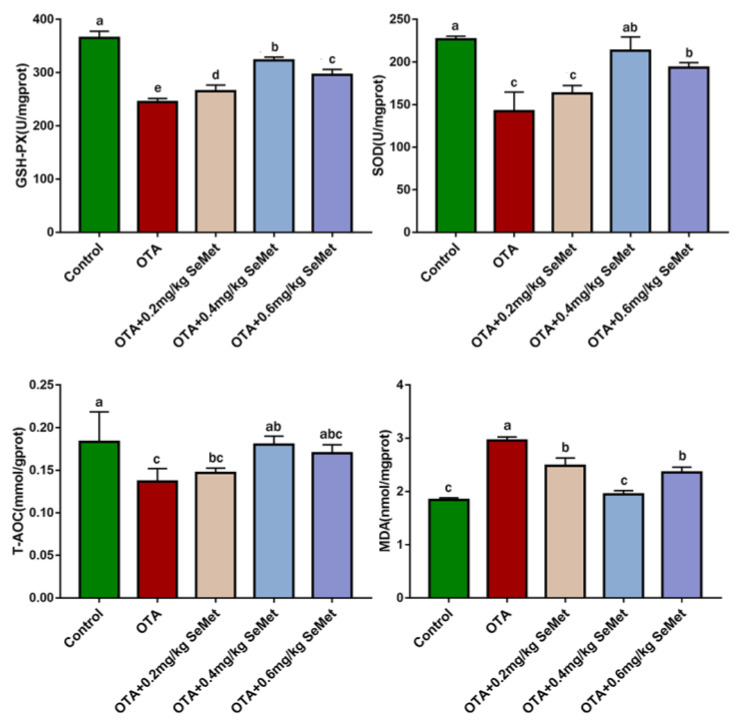
Effects of SeMet on oxidative stress markers in rabbits after OTA treatment (Mean ± SD, *n* = 6). ^a–e^ Means column carrying different superscript letters and indicates significant differences (*p* < 0.05).

The ROS content in rabbit liver is shown in Figure 5. The results showed that the ROS level in OTA-treated group was significantly higher than that in the control group (*p* < 0.05). After SeMet pretreatment, ROS content decreased to different degrees and decreased by 30.2% in the 0.4 mg/kg SeMet + OTA group (*p* < 0.05), which was significantly different from that in OTA-treated group. The levels of T-AOC, SOD, GSH-Px and MDA in the liver tissues of each group were detected by corresponding kits. As shown in Figure 6, compared with the control group, the enzyme activities of GSH-Px, SOD and T-AOC in the OTA treatment group were extremely decreased (*p* < 0.05), and MDA content was extremely significantly increased (*p* < 0.05). After SeMet pretreatment, the levels of T-AOC, SOD and GSH-Px increased to different degrees compared with the OTA-treated group, while the levels of MDA decreased to different degrees. Among them, the 0.4 mg/kg SeMet + OTA group showed the most significant improvement. Compared with OTA-treated group, the activities of GSH-Px, SOD and T-AOC were increased by 31.83%, 49.76% and 31.7% (*p* < 0.05), and the content of MDA was decreased by 34.23% (*p* < 0.05).

### 2.4. SeMet Modulated Nrf2 and HO-1 Expression in OTA-Induced Rabbit Hepatic Toxicity

**Figure 7:**
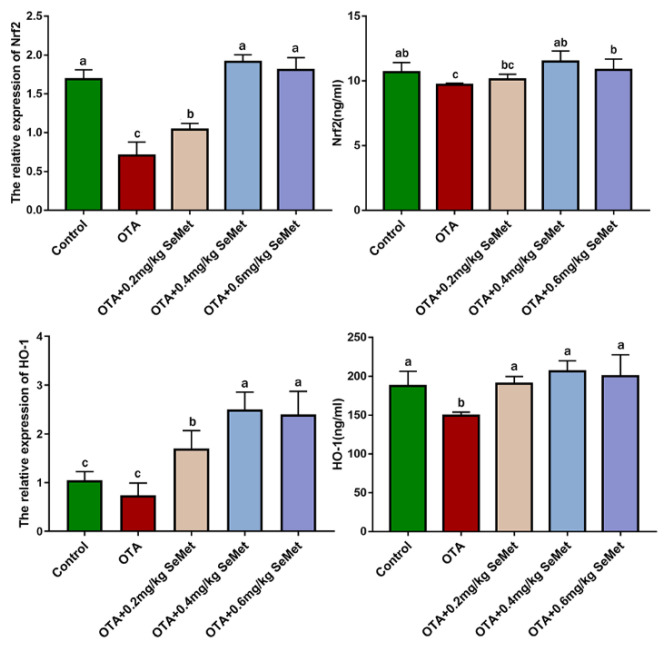
Nrf2 and HO-1 protein and mRNA expression (Mean ± SD, *n* = 6). ^a–c^ Means column carrying different superscript letters and indicates significant differences (*p* < 0.05).

The mRNA and protein expression changes of Nrf2 and HO-1 in rabbits are shown in Figure 7. Compared with the control group, the mRNA expression levels of Nrf2 and HO-1 in the OTA-treated group were significantly decreased by 58.1% and 49.65% (*p* < 0.05), respectively, and the protein expression levels were significantly decreased by 9% (*p* < 0.05) and 20.28%. Compared with the OTA-treated group, Nrf2 and HO-1 protein and mRNA levels were increased to varying degrees after SeMet pretreatment. After 0.4 mg/kg SeMet pretreatment, the mRNA expression levels of Nrf2 and HO-1 were significantly increased by 1.7 times and 1.08 times (*p* < 0.05), respectively, and the protein expression levels were significantly increased by 18.54% and 38.05% (*p* < 0.05), respectively, compared with the OTA-treated group.

### 2.5. SeMet Improves Rabbit Hepatic Inflammation Induced by OTA

**Figure 8:**
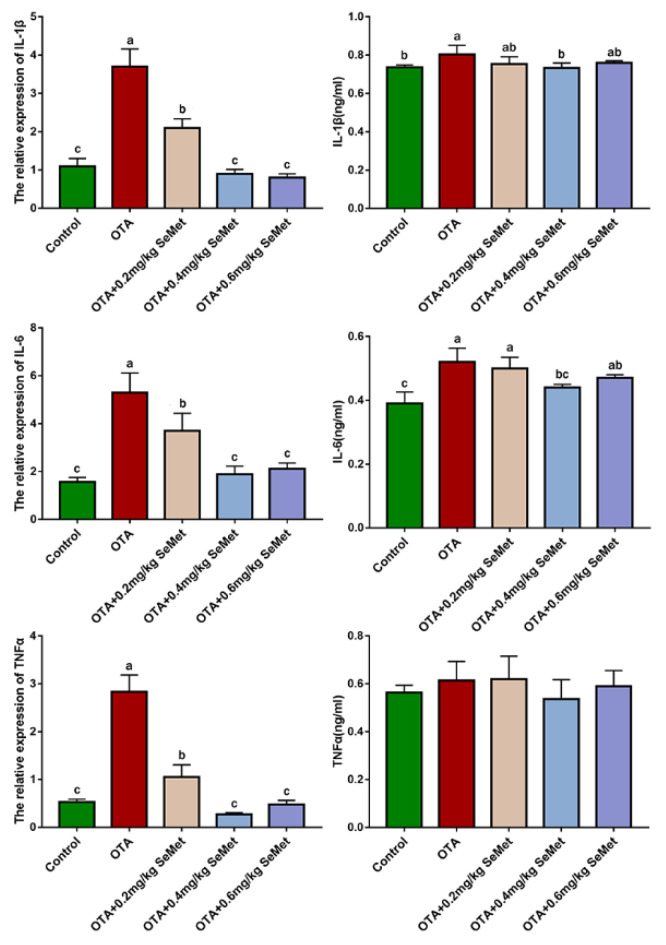
Inflammatory indexes protein and mRNA expression (Mean ± SD, *n* = 6). ^a–c^ Means column carrying different superscript letters and indicates significant differences (*p* < 0.05).

The indexes of rabbit liver inflammation in rabbits are shown in Figure 8, and the mRNA and protein expressions of each index had the same trend. Compared with the control group, the mRNA expression levels of IL-1β, IL-6 and TNF-α in the OTA-treated group were significantly increased (*p* < 0.05), while the protein expression levels were increased by 9%, 33.3% (*p* < 0.05) and 8.87%, respectively. After SeMet pretreatment, the protein and mRNA levels of IL-6, IL-1β and TNF-α were decreased to varying degrees compared with the OTA-treated group. Compared with the OTA-treated group, the mRNA expression levels of IL-1β, IL-6 and TNF-α in the 0.4 mg/kg SeMet group were significantly decreased by 75.67%, 64.34% and 90.46% (*p* < 0.05), respectively. The protein expression level was decreased by 8.71%, 15.38% (*p* < 0.05) and 1.25%, respectively.

## 3. Discussion

OTA has a highly toxic effect on both humans and animals, and its toxic mechanism may be related to oxidative stress induction, apoptosis induction, inhibition of protein synthesis, interference of cell signal transduction, etc. SeMet is widely used as a feed additive due to its antagonistic effect against various mycotoxins, excellent antioxidant capacity, high bioavailability and low toxicity [22]. Results from previous studies suggest that ingestion and accumulation of OTA at lower doses can cause organ damage in the body. Additionally, the protective effect of Se alone in the diet on animals was confirmed [23,24]. However, the protective effect of Se on OTA-induced liver injury has not been reported. The purpose of this experiment was to explore the protective effect of SeMet on OTA-induced liver injury in rabbits and to preliminarily explore the protective effect of different doses of SeMet. Therefore, we did not examine the effect of SeMet alone. 

The liver, as a detoxifying organ, is one of the target organs of OTA toxicity. Changes in serum ALT, AST and ALP levels are usually used as important indicators to judge whether liver function is normal [25]. Previous studies have shown that OTA exposure can increase serum ALT, AST and ALP levels in rats, broilers and other animals. This is consistent with the results of this study. In this study, the enzyme activities of AST, ALT and ALP, which are important indexes of liver function, were significantly increased in the serum of the OTA treatment group, suggesting that liver cells were damaged. The reason may be that OTA plays a toxic role in liver cell necrosis. Studies have shown that 1% liver cell necrosis can double the activity of ALT enzyme in the blood and significantly increase the levels of AST and ALP [26]. When cells are damaged or cell membrane permeability increases, excessive release of diseased liver cells or biliary obstruction will increase the levels of ALT, AST and ALP in the blood [27,28]. These findings are supported by liver histomorphology. Sara et al. found that OTA caused multifocal lymphoplasmacellular hepatitis, periportal fibrosis and necrosis observed in rat liver [29]. G Aydin et al. also observed granular or vacuolar degeneration and necrosis of liver cells, dilation of hepatic sinusoids and central veins, hyperplasia of bile ducts and mild fibrous tissue hyperplasia in rats 30 days after OTA administration at 289 μg/kg [30]. The main pathogenesis of hepatic fibrosis is the activation of dormant hepatocytes, which secrete a large amount of extracellular matrix deposited in the liver parenchyma after long-term injury by various injury factors. Chronic inflammation can also cause fibrous connective tissue hyperplasia, leading to a pathological liver fibrosis state [31]. In this study, we observed that the OTA treatment group had liver cell disorder, severe cytoplasmic vacuolation, a large number of red blood cells in the hepatic sinusoidal space, reduced liver glycogen content and significantly aggravated liver fibrosis, which was consistent with the lesions previously reported. OTA exposure may lead to liver disease injury in animals.

Se is an essential trace element for biological growth. It has the biological functions of improving immunity, anti-oxidation, anti-inflammation and promoting animal growth and reproduction. After feeding SD rats with Se-enriched tea, the phagocytic cells of experimental rats increased to 6.2 times that of the control group, and the phagocytic index reached 2.1 times that of the control group, suggesting that Se can significantly improve the non-specific immunity of SD rats [32]. Our previous studies also showed that dietary supplementation of SeMet can reduce the elevated levels of AST, ALT and ALP enzymes induced by the T-2 toxin and protect the liver from damage [9]. In our study, when rabbits were pretreated with 0.4 mg/kg SeMet, the activity levels of AST, ALT and ALP enzymes were significantly decreased, which was close to the control group. The liver glycogen synthesis ability was significantly improved, the degree of liver fibrosis was significantly reduced and histopathological changes were also significantly improved. These results suggest that 0.4 mg/kg SeMet has a protective effect on OTA-induced liver injury. 

A large number of studies proved that oxidative stress is one of the main mechanisms of OTA toxicity. OTA exposure leads to the overproduction of free radicals and induces oxidative stress in the body [33]. Studies have shown that OTA exposure can lead to a dependent increase in ROS concentration in primary rat PT cells and LC-PK1 cells [34]. Excessive accumulation of oxidants such as ROS in the body exceeds the scavenging ability of the body, leading to significantly increased lipid peroxidation levels in various tissues, resulting in DNA damage and abnormal protein expression, thus causing body damage [35]. Enrique et al. showed that OTA could induce ROS increase in HepG2 cells in a time-dependent manner [36]. When a certain amount of OTA enters the body, the antioxidant defense system in the body is destroyed, leading to a significant increase in ROS and MDA levels and a significant decrease in SOD, GSH-Px and T-AOC levels. As one of the earliest metabolic organs, the liver can oxidize, reduce or hydrolyze toxic substances generated by external or internal metabolism, and convert them into non-toxic substances or substances with large solubility, which is discharged with bile and urine. In this process, a large number of superoxide anion radicals are produced. As a result, many antioxidant enzymes in the liver, such as SOD and GSH-Px, are highly active. The liver is extremely vulnerable to oxidative damage when exposed to high concentrations of exotic organisms and other chemicals [37]. MDA level is a marker of lipid peroxidation and indirectly reflects the degree of cell damage [38]. 

**Figure 9:**
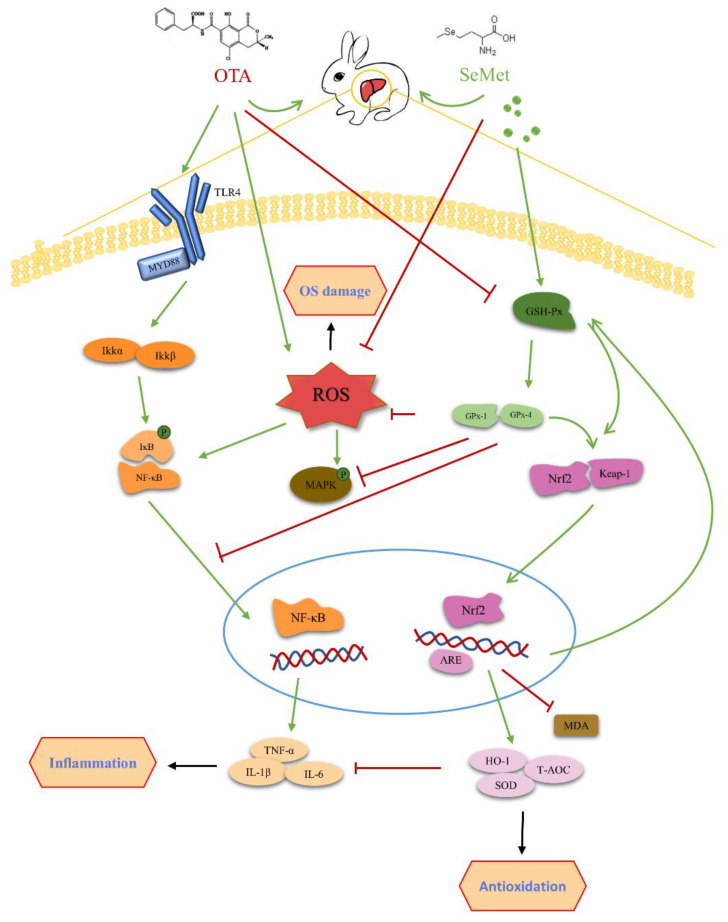
Schematic diagram of the mechanism of SeMet improving the liver damage induced by OTA poisoning by activating Nrf2 and inhibiting the NF-κB pathway.

Nrf2 is a transcription factor responsible for the regulation of mammalian cell REDOX balance, a protective antioxidant, and stage II detoxification. Under physiological conditions, Nrf2 binds to Kelch-like Ech-associated protein 1 (KEAP-1) and exists in the cytoplasmic matrix. When ROS accumulates in the body, Keap-1/Nrf2 binding is destroyed, and Nrf2 is activated into the nucleus and binds with antioxidant reaction elements (ARE) to activate transcription and expression of downstream antioxidant enzymes (SOD, GSH-Px, etc.) [39]. HO-1, NQO1, GST and other downstream target genes of Nrf2 are involved in the degradation of pre-oxidant heme, and their products are proven to avoid oxidative damage of cells, reduce the inflammatory response, regulate apoptosis, promote angiogenesis and so on [40] (Figure 9). Nrf2/HO-1 was shown to play a protective role in burn-induced oxidative liver damage, CCL4-induced liver damage, nickel-induced DNA methylation and inflammation [41,42]. Multiple studies have shown that OTA inhibits the Nrf2 oxidative stress response pathway [43,44]. OTA inhibition of Nrf2 activation and gene transcription, as well as OTA-induced Nrf2 protein loss, force cells to lose the ability to defend against physiological and compound-induced oxidative stress [11]. Inhibition of Nrf2 can reduce glutathione synthesis, oxidative glutathione cycling and Oxidoreductase activity, making cells and tissues more susceptible to oxidative stress. Cavin et al. showed that OTA inhibited the protein expression of Nrf2 and its regulatory genes in rat liver and kidney cells and reduced the expression levels of core genes Nrf2 and HO-1 [45]. Nrf2 knockout animals showed higher sensitivity to various chemical damage [10]. Our study showed that after OTA gavage for 7 days, ROS content in the liver significantly increased, MDA level of lipid peroxidation product significantly increased, t-AOC and activities of antioxidant enzymes SOD and GSH-Px significantly decreased and Nrf2 mRNA and protein expression in the liver was significantly lower than in the control group. Nrf2-regulated ho-1 expression was also significantly lower than in the control group. It indicates that OTA induces excessive ROS production in the liver and inhibits the antioxidant defense system of the body, resulting in the imbalance of the oxidation/antioxidant system, thus causing oxidative stress in liver cells.

Se is an excellent antioxidant, which can protect the activities of various antioxidant enzymes and also play an antioxidant role by forming selenoproteins. In a rat retinal ischemia-reperfusion experiment, Se upregulated SOD, GSH and T-AOC levels, decreased MDA expression and enhanced antioxidant capacity, thereby limiting ischemia-induced retinal thickening and apoptosis [46]. Guo et al. [47] reported that SeMet could improve GSH-Px activity and T-AOC levels in the serum and liver of weaned piglets, reduce MDA content, enhance the antioxidant capacity of weaned piglets and promote their growth. Yao [48] reported that after Se pre-incubated grass carp liver and kidney cells, the fine Nrf2/Keap1 signaling pathway was activated, and the activity of intracellular antioxidant enzymes was enhanced to protect the cell oxidative damage induced by sodium nitrite. Our previous studies have also shown that dietary Se supplementation reduces T2 toxin-induced oxidative damage in rabbit kidneys [49]. However, previous studies have also shown that Se supplementation can affect its biological characteristics. There is a safe limit between the optimal supplemental level of Se and the toxic dose. This experiment found that 0.4 mg/kg SeMet can significantly reduce OTA-induced ROS and MDA contents and significantly increase the activities of SOD, GSH-Px and T-AOC levels after adding different doses of SeMet to the diet of rabbits. The mRNA and protein expressions of Nrf2 and HO-1 were also significantly increased, indicating that 0.4 mg/kg SeMet reduced the level of oxidative stress after OTA poisoning in rabbits. However, with the increase in SeMet dose, ROS and MDA expression levels did not further decrease after 0.6 mg/kg SeMet pretreatment, but SOD, GSH-Px activities and T-AOC levels decreased. This may be related to the toxic effect of excessive Se. Studies have shown that excessive Se supplementation can have toxic effects on the body. These include genotoxicity, embryological toxicity, reproductive toxicity, immunotoxicity and cytotoxicity, and they can even lead to death in severe cases [50]. The toxic mechanism of Se may involve oxidative stress, inhibition of biofilm formation and interference with enzyme activity [51]. When the Se intake dose is too high, it will inhibit the methylation metabolism of Se in the form of selenocysteine and force Se to enter the REDOX process, produce superoxide, cause oxidative stress reaction, generate free radicals, inhibit the function of important enzymes and proteins and also cause lipid peroxidation of biofilms [52]. Wu et al. [53] found that after Se poisoning in ducklings, with the prolongation of the test time, the levels of free radicals and hepatocyte apoptosis showed a trend of gradual increase, and Se poisoning could induce cell apoptosis through free radicals and produce toxic effects. The toxicity of Se also varies with animal species, nutritional status and the route of administration. At present, it has not been studied how much dose of Se can best balance the protective and toxic effects of Se in rabbit feed, and further research and exploration are required. The results show that the oxidative stress level of the liver increased with the rise of the SeMet dose. These results suggest that 0.4 mg/kg SeMet may play a protective role in OTA-induced liver injury through its antioxidant capacity.

Studies have shown that oxidative stress is closely related to inflammation. Under oxidative stress, the body produces a large number of reactive oxygen species, which activate MAPK and NF-κB inflammatory signaling pathways and increase the expression of pro-inflammatory factors [54]. Oxidative stress affects the body’s immune system and inflammatory response [55]. NF-κB, as a multidirectional and pluripotent regulatory factor, is the core of inflammation and anti-inflammatory activity, and its activation is mainly determined by phosphorylation of IκB [56]. The combination of NF-κB and IκB is static in a normal physiological state. When the organism is stimulated by external factors, the increase in ROS level caused by OTA in Ju’s study will stimulate the TLR4/MYD88 signaling pathway [57]. IκB kinase (IKK) is activated first when the receptor protein is stimulated. Ubiquitination and proteasome degradation of IκB allows NF-κB to be released from the cytoplasm to the nucleus to initiate gene expression [58]. Activation of NF-κB activates the expression of various inflammatory and adhesion factors, leading directly to inflammation [59], which leads to liver damage (Figure 9). Inflammation is an important indicator of liver injury caused by toxic physical or chemical stimuli. Inflammation plays an important role in the repair of liver injury [60]. TNF-α is the first inflammatory factor produced in the inflammatory response, which can induce the production of other cytokines, affect NF-κB, a key factor in regulating inflammatory response, and promote the production of free radicals, magnifying the inflammatory response and aggravating liver injury [61]. During liver injury, inflammatory cells are activated, and inflammatory cytokines (TNF-A, IL-1β and IL-6) are released, which are responsible for the accumulation of neutrophils in the liver, ultimately leading to increased cytokine expression [62]. Studies have shown that mycotoxins, including OTA, can promote the production of pro-inflammatory factor TNF-α in rats [63], and mild OTA poisoning can lead to a persistent systemic inflammatory response in pigs [64]. Since 0.4 mg/kg SeMet significantly reduced OTA-induced oxidative stress, we examined the expression of three pro-inflammatory cytokines, IL-6, TNF-α and IL-1β, to further explore the protective mechanism of SeMet. The results showed that different doses of SeMet decreased the expression of IL-6, TNF-α and IL-1β mRNA and protein, suggesting that SeMet alleviates OTA-induced liver injury by inhibiting the inflammatory response. This may be related to Se being an important component of selenate. Studies have shown that Se can reduce OTA-induced renal toxicity in pigs by increasing the expression and release of GPx1 and GPx4 [65]. Selenoproteins help control the overproduction of free radicals in inflammatory sites, and Se supplementation is associated with metabolism and the regulation of inflammatory cytokines [66]. In the process of wound healing, Se can increase Nrf2 protein content, reduce KEAP1 expression, activate the Nrf2-ARE signaling pathway and promote GPX-1, GPX-4, selenoprotein S, selenoprotein P and other selenoproteins. Additionally, in wound healing, the inflammatory stage shows a variety of reactions. Such as antioxidant activity, inhibition of inflammatory cytokines, scavenging of peroxynitrite (a kind of supercritical ion) and inhibition of NF-κB signaling and MAPK protein activity [67,68,69]. In addition, Se can activate the Nrf2 transcription factor in mycotoxin infection, which further upregulates the HO-1 gene downstream of Nrf2. Studies have shown that HO-1 has immunomodulatory and anti-inflammatory effects [70]. HO-1 has a protective effect on acute or chronic liver injury, including hepatitis and cirrhosis [71]. In this study, we found that adding 0.4 mg/kg SeMet to the diet reduced OTA-induced pathological liver damage and reduced oxidative stress and inflammatory responses in the liver. It may be that SeMet protects the liver and reduces OTA-induced liver damage by activating the Nrf2/HO-1 signaling pathway, eliminating ROS, reducing oxidative stress and promoting the expression of Se proteins such as GPX-1 and GPX-4.

## 4. Conclusions

In conclusion, we confirmed that OTA could inhibit the Nrf2 transcription factor, increase oxidation products, reduce liver antioxidant capacity, induce liver inflammation and cause liver injury. Medium dose SeMet has an obvious protective effect on OTA-induced liver injury in rabbits, and its protective mechanism may be related to the activation of the Nrf2 transcription factor and further activation of HO-1. This study provides a theoretical basis for SeMet to prevent OTA poisoning in rabbits.

## 5. Materials and Methods

### 5.1. Animals and Treatments

Our animals were housed in individual cages and allowed to acclimate to a 12/12 h light/dark cycle in a temperature and humidity-regulated area. The animals were free to help themselves to food and water. Each rabbit was acclimated to the facility for at least 7 days before drug administration. Only healthy animals within the specified weight range and animals with no obvious clinical signs of disease or deformity were included in the study. A total of 60 healthy rabbits aged 35 days were randomly divided into 5 groups with 12 rabbits in each group (*n* = 12): Control group, OTA-treated group (0.2 mg/kg OTA), OTA + 0.2 mg/kg SeMet (0.2 mg/kg OTA + 0.2 mg/kg Se), OTA + 0.4 mg/kg SeMet (0.2 mg/kg OTA + 0.4 mg /kg Se) and OTA + 0.6 mg/kg SeMet (0.2 mg/kg OTA + 0.6 mg/kg Se). Rabbits in the control group and OTA-treated group were fed a normal diet, and rabbits in other groups were fed diets supplemented with 0.2 mg/kg, 0.4 mg/kg and 0.6 mg/kg SeMet (dissolved in distilled water, evenly sprayed into the basic diet and then dried for reserve), respectively [9]. From the 15th day, the control group was gavaged with 0.5 mL NaHCO_3_ solution, and the other groups were gavaged with 0.5 mL NaHCO_3_ solution containing OTA (0.2 mg/kg OTA, Pribolab) for a week [72,73]. The study was approved by the Institutional Animal Care and Use Committee of Henan University of Science and Technology (China) (No.20190619024) on 19 June 2019. The compositional and chemical analysis of the basic diet is shown in Table 1.

### 5.2. Sample Preparation

The experiment lasted for 21 d, and 30 rabbits (6 rabbits in each group were randomly selected) were euthanized after fasting for 24 h after the last feeding (*n* = 6). Blood and liver samples were collected. Part of the liver samples was fixed in 4% paraformaldehyde, and the rest were immediately stored at −80 °C for further experiments. We applied Emla 5% cream (AstraZeneca, Cambridge, UK) to the skin covering the vein at the edge of the ear about 5 min before the rabbit was euthanized and warmed the ear by gentle stroking. Five minutes later, pentobarbital was slowly injected into the marginal auricular vein of rabbits at a dose of 100 mg/kg. All animal care and handling were carried out in adherence to institutional and national guidelines.

### 5.3. Biochemical Assays

Blood samples were centrifuged at 3500 rpm for 10 min at 4 °C, and plasma was isolated immediately after collection. The plasma samples were stored at −20 °C, and the activities of AST, ALT and ALP in the plasma were measured by the Excellence 300 automatic biochemical analyzer (Shanghai Kehua, Shanghai, China). The kit was purchased from Shanghai Kehua Biological Engineering Co., Ltd. (Shanghai, China).

### 5.4. Histopathological Analysis

After euthanasia, liver tissue samples were obtained aseptically and fixed in 4% paraformaldehyde. Then, 48 h later, the tissues were dehydrated, embedded in paraffin, sliced into 5 μm sections and stained with hematoxylin and eosin (H.E) for histological examination. Subsequently, the changes in liver glycogen content were determined by PAS staining, and the degree of liver fibrosis was determined by MASSON staining. Three representative sections were taken from each liver.

### 5.5. Measurement of ROS

DHE stainings were performed as previously described. Frozen liver sections were stained with DHE (10 mM) at 37 °C for 30 min. Fluorescence was visualized by confocal microscopy (LSM 710, Zeiss, Jena, Germany). The fluorescence intensity of DHE staining was quantitatively analyzed by ImageJ software (version 1.47; National Institutes of Health, Bethesda, MD, USA).

### 5.6. Measurement of Oxidative Stress Markers

The liver tissue was homogenized by adding physiological saline 1:9, centrifuged at 3000 rpm/min for 15 min, and the supernatant was collected. The levels of MDA, SOD, T-AOC and GSH-PX were tested using a specific test kit (Nanjing Jiancheng Institute of Bioengineering, Nanjing, China) according to the manufacturer’s instructions.

### 5.7. Quantitative Real-Time PCR Analysis

Total RNA in jejunum tissue was extracted using TRIzol reagent (CW0580; CoWin Biosciences Co., Ltd., Beijing, China), and cDNA was generated using a reverse transcription kit (RR036A; Takara, Kusatsu, Japan). The SYBR^®^Premix Ex Taq™ kit (Takara, Dalian, China) was used for quantitative PCR analysis. The expression levels of the target gene were evaluated using the comparative 2−ΔΔCtmethod. The PCR primer sequences are shown in Table 2.

### 5.8. Enzyme-Linked Immunosorbent Assay

The enzyme-linked immunosorbent assay (ELISA) was used to detect the level of related proteins in rabbit liver tissue. The tissue sample was separated, centrifuged and added to the sample dilution. The tissue sample was analyzed using rabbit ELISA kits (Myhalic Biotechnological Co., Ltd., Wuhan, China) according to the manufacturer’s instructions. The optical density (OD) was measured using a microplate reader (Bio-Rad, Hercules, CA, USA) at a wavelength of 450 nm.

### 5.9. Statistical Analysis

All data were examined for normality and homogeneity by the Shapiro–Wilks test. Data analysis was performed using SPSS 20.0 software, and the data were expressed as mean ± standard deviation (SD). Differences between groups were compared using one-way analysis of variance (ANOVA) and Duncan’s multiple comparison test. The difference between groups was considered significant when the probability (*p*) was <0.05.

## Figures and Tables

**Table 1:** Ingredients and chemical composition of the basal diets.

Feed Ingredients	Percent %	Nutritional Level	Percent %
Corn	19	Crude Protein	≥17
Soya bean meal	19	Crude Fiber	≤20
Wheat bran	18	Crude ash	≤12
PeanutHull	14	Calcium	≤12
Bean straw	10	phosphorus	≥0.55
wormwood rod	7	sodium chloride	0.3–1.2
vinasse	6	lysine	≥0.65
premix	4	moisture	≤12.5
soybean oil	3	digestible energy (MJ/kg)	9.37

Note: The premix provided the following per kg of diet: vitamin A 10,000 IU; vitamin D3, 1800 IU; vitamin E, 15 mg; vitamin K3, 4.5 mg; vitamin B1, 0.5 mg; vitamin B2, 4 mg; vitamin B12, 0.001 mg; folic acid, 0.1 mg; pantothenic acid, 7 mg; nicotinic acid, 20 mg; I, 1 mg; Mn, 60 mg; Cu, 5.5 mg; Zn, 75 mg; Fe, 40 mg; Co, 0.3 mg; Se, 0.08 mg. Values were calculated from the Chinese feed database provided with tables of feed composition and nutritive values in China (31st edition).

**Table 2:** Primer sequences used in RT-PCR.

Gene	Primer Sequences (5′ to 3′)	Product Length (bp)
β-actin	CGTGCGGGACATCAAGGAG	177
	AGGAAGGAGGGCTGGAAGAG	
Nrf2	CTCCATATCCCATTCCCTGTA	148
	TCTGAGCAGCCACTTTATTCT	
HO-1	CAGGTGACTGCCGAGGGTT	100
	GACCGGGTTCTCCTTGTTGTG	
IL-1β	AAGACGATAAACCTACCCTGC	121
	GACTCAAATTCCAGCTTGTCC	
IL-6	GAAGACGACCACGATCCAC	115
	GCCCATGAAATTCCGCAAG	
TNF-α	AAGAGTCCCCAAACAACCTCC	122
	CTCCACTTGCGGGTTTGCTAC	

## Data Availability

Not applicable.
